# Association between mass media exposure and cervical cancer screening uptake among women aged 30–49 years in four sub-Saharan African countries: a pooled analysis of demographic and health surveys

**DOI:** 10.1186/s12905-025-04107-1

**Published:** 2025-12-22

**Authors:** Chrispin Mandiwa, Mattia Sanna, Wayne Gao

**Affiliations:** 1 Ph.D. Program in Global Health and Health Security, College of Public Health, Taipei Medical University, Taipei, Taiwan; 2 Master’s and Ph.D. Program in Global Health and Health Security, College of Public Health, Taipei Medical University, Taipei, Taiwan; 3 Project Hope, Lilongwe, Malawi

**Keywords:** Cervical cancer, Screening, Media exposure, Women, Sub-Saharan africa

## Abstract

**Background:**

Cervical cancer remains a major public health challenge in sub-Saharan Africa (SSA), mainly due to low screening uptake. Mass media exposure (including radio, newspapers, and television) can play a key role in promoting health services utilisation; however, little is known about its relationship with cervical cancer screening uptake in SSA. This study examined the association between mass media exposure and cervical cancer screening uptake among women aged 30–49 years in four SSA countries.

**Methods:**

This cross-sectional study utilized data from the Demographic and Health Surveys conducted between 2022 and 2023 in four SSA countries: Ghana, Kenya, Mozambique, and Tanzania. A pooled weighted sample of 26,936 women aged 30–49 years was analysed. Univariable and multivariable logistic regression models were fitted to assess the association between mass media exposure and cervical cancer screening uptake. Adjusted odds ratios with their corresponding 95% confidence intervals were estimated.

**Results:**

The pooled prevalence of cervical cancer screening was 15.2% (95% CI: 14.5–15.9), with the lowest rate in Ghana at 7.3% and highest in Kenya at 27.0%. The multivariable analysis revealed that women exposed to mass media were 74% more likely to have been screened for cervical cancer (AOR: 1.74; 95% CI: 1.40–2.16.) compared to those without media exposure.

**Conclusion:**

This study demonstrated a positive association between mass media exposure and uptake of cervical cancer screening among women in SSA. This highlights the important role that mass media can play in promoting the uptake of screening in the region. Thus, increasing access to media platforms, such as radio, television, and newspapers could enhance awareness and participation in cervical cancer screening services, ultimately helping reduce the disease burden in SSA.

**Supplementary Information:**

The online version contains supplementary material available at 10.1186/s12905-025-04107-1.

## Background

Cervical cancer is the abnormal growth of cells in the lining of the cervix. It is primarily caused by persistent infection with high-risk strains of human papillomavirus (HPV), particularly types 16 and 18, which are common sexually transmitted infections [1–3].

Cervical cancer remains a significant public health challenge, particularly in low- and middle-income countries (LMICs). In 2022, an estimated 662,301 new cases and 348,874 deaths were reported worldwide, with 94% of these deaths occurring in resource-limited settings [4]. That same year, sub-Saharan Africa (SSA) reported the highest regional burden of cervical cancer, with an estimated age-standardized incidence rate (ASIR) of 26.4 per 100,000 women and an estimated age-standardized mortality rate (ASMR) of 17.6 per 100,000 women. These figures were significantly higher than those reported in the United States, where the ASIR and ASMR in 2022 were 6.3 and 2.2 per 100,000 women, respectively [5]. These alarming statistics highlight the severe impact of the disease on women’s health and underscore the urgent need for targeted interventions to enhance screening, early detection, and timely treatment to reduce the disease burden in SSA.

To address the high burden of cervical cancer, the World Health Organization (WHO) released the Global Strategy to Accelerate the Elimination of Cervical Cancer in 2020, aiming to reduce the incidence rate to below 4 per 100,000 women annually in all countries within a century [6]. To achieve this goal, the WHO set the following 90-70−90 targets to be met by 2030: vaccinating 90% of girls with the HPV vaccine by the age of 15 years; screening 70% of women by the age of 35 years and again by 45 years of age; ensuring that 90% of women with a positive screening result or cervical lesion receive appropriate treatment [6]. In 2021, the WHO also issued updated cervical cancer screening guidelines recommending that women aged 30–49 years be screened at intervals of 5 to 10 years using HPV DNA testing, or every 3 years using visual inspection with acetic acid (VIA) or cytology [7]. This screening approach is crucial for the early detection and timely management of cervical cancer, which can prevent most cases [8, 9]. Evidence shows that well-implemented screening programs can reduce cervical cancer incidence and mortality by up to 80% [9], highlighting the critical role of screening in reducing the disease burden.

Despite these recommendations, the uptake of cervical cancer screening in SSA remains low, with only 10.3% of women aged 15 to 49 years reported to have ever been screened [10], compared to over 60% in high-income countries [11]. This low uptake is largely due to barriers such as limited access to healthcare facilities [12–14], long distances to health facilities [15–17], and lack of awareness about cervical cancer and screening methods among women [18–21].

Exposure to mass media (such as radio, newspapers, and television) is widely recognised for raising awareness of diseases and preventive measures [22–24]. Studies have shown that exposure to mass media can significantly affect health-seeking behaviour, such as promoting vaccination during public health emergencies [25, 26], encouraging young women to test for HIV [27], increasing the uptake of antenatal care services [28–30] and contraceptive use [31–33].

Given the widespread reach of mass media and its ability to raise awareness, especially in settings with limited healthcare access, leveraging it could offer a low-cost, scalable strategy to address informational barriers and improve screening uptake. Nonetheless, the role of mass media exposure in promoting cervical cancer screening has not been extensively investigated in SSA. Most existing studies have been limited to single country or subnational analyses and have not focused on the WHO-recommended target group of women aged 30–49 years [34–36]. This study aimed to address this gap by examining the association between mass media exposure and cervical cancer screening among women aged 30–49 years in four SSA countries using recent Demographic and Health Survey (DHS) data. Understanding this association is crucial for developing effective interventions that leverage mass media to improve screening uptake, thereby contributing to the achievement of the WHO 90-70−90 targets, and ultimately reduce the cervical cancer burden in the region.

## Methods

### Study design, setting, and data source

Cross-sectional data from the DHS conducted between 2022 and 2023 in four SSA countries, namely Tanzania, Mozambique, Kenya and Ghana, were analysed in this study. These countries were selected because they represent different regions of SSA (East, West, and Southern Africa) and have the most recent DHS data with key variables of interest, including mass media exposure, socio-demographic factors, and health seeking behaviours such as HIV testing.

### Brief overview of the DHS

The DHS are nationally representative household surveys conducted approximately every five years in many LMICs. They collect standardised data on various indicators such as maternal and child health, family planning, HIV/AIDS, malaria, and nutrition to support evidence-based policymaking and program development. The data are gathered through a combination of face-to-face interviews and biomarker collection, providing a comprehensive assessment of population health.

### Sampling technique and study participants

**Fig. 1:**
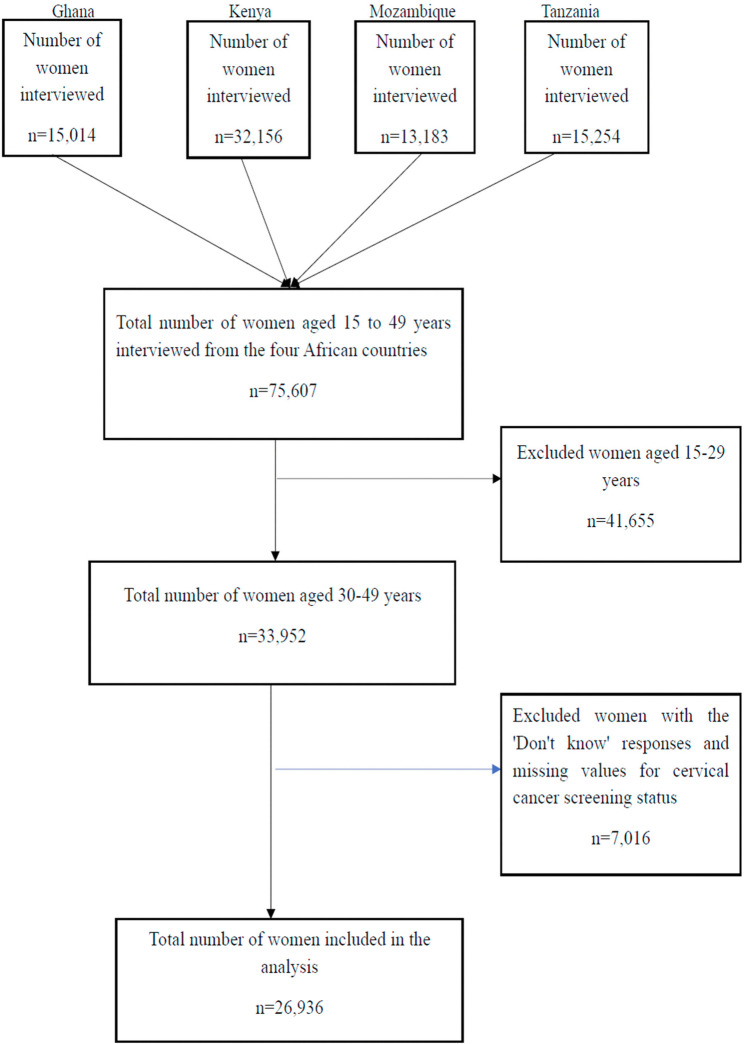
Flow diagram summarizing the process of selecting the sample included in the study

The DHS employs a stratified, two-stage cluster sampling design. The first stage involves the selection of Enumeration Areas (EAs) using a probability proportional to size method. The second stage involves the selection of households within each randomly selected EA. All women aged 15–49 years in the selected households are eligible for individual interviews. The DHS uses four questionnaires to collect data: the Women’s Questionnaire, the Men’s Questionnaire, the Household Questionnaire, and the Biomarker Questionnaire. For this study, we used data collected with the Women’s Questionnaire, focusing on women aged 30–49 years. A total of 26,936 participants were included in this study. The sample selection process is shown in Fig. 1.

### Conceptual framework

**Fig. 2:**
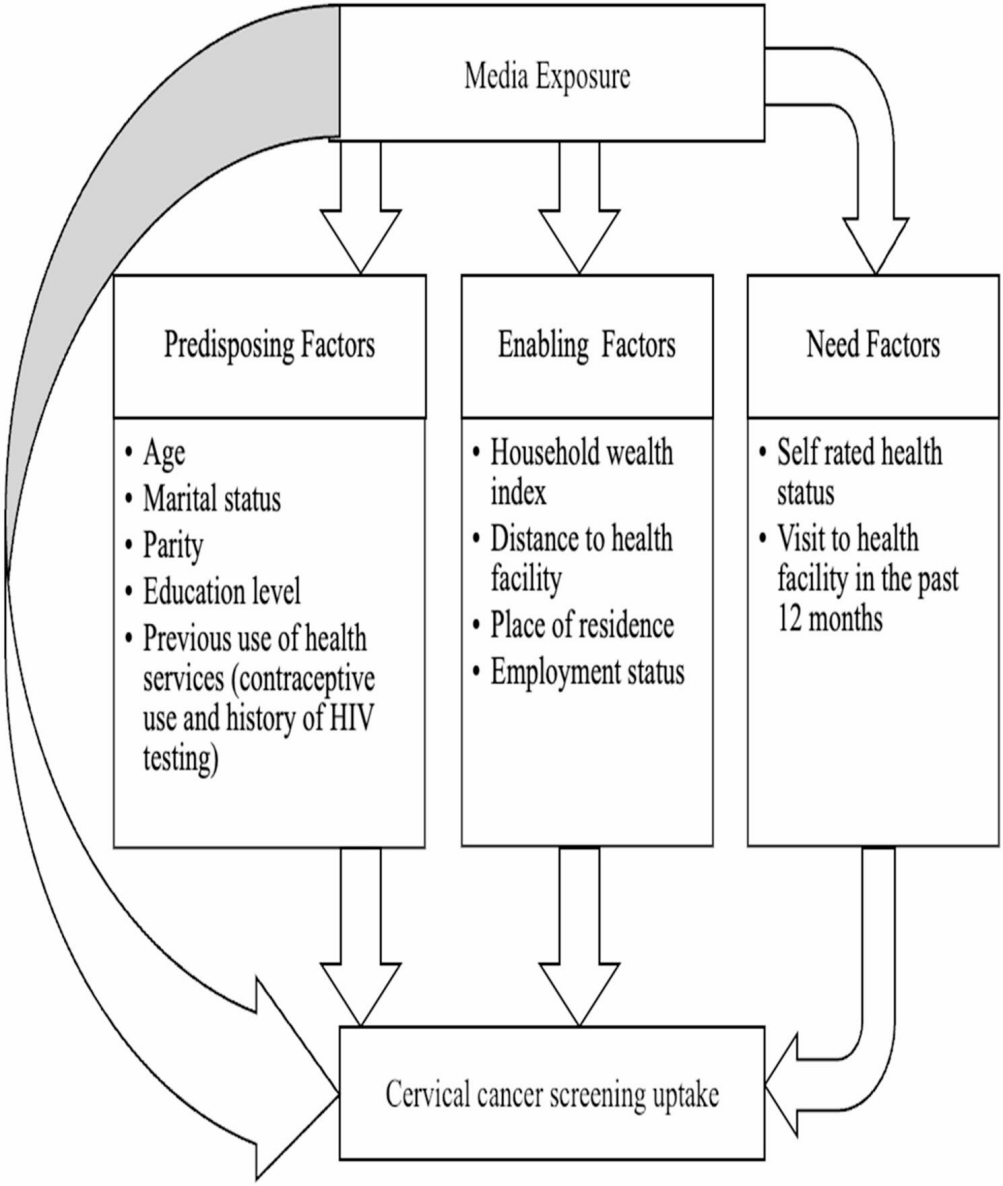
Conceptual framework adapted from Andersen’s Behavioural Model of Health Services Use

To guide our analysis, we adapted Andersen’s Behavioural Model of Health Services Use, which posits that healthcare utilization is influenced by predisposing, enabling and need factors [37, 38]. In this context, the model suggests that mass media exposure may directly influence screening uptake or operate indirectly through its effects on predisposing factors (e.g., demographic and behavioural characteristics), enabling factors (e.g., access and logistical conditions), and need factors (e.g., perceived health status) as displayed in Fig. 2.

### Study variables and measurements

#### Outcome variable

The outcome variable for this study was cervical cancer screening, which was treated as a binary outcome. Women were asked, ‘Has a doctor or other healthcare worker ever tested you for cervical cancer?’ The responses to this question were ‘Yes,’ ‘No,’ and ‘Don’t know.’ We excluded the ‘Don’t know’ responses and women with missing values for cervical cancer screening status.

#### Exposure variable

The exposure variable of interest was self-reported mass media exposure. Women were asked the following questions: (1) Do you read a newspaper or magazine at least once a week, less than once a week, or not at all? (2) Do you listen to the radio at least once a week, less than once a week, or not at all? (3) Do you watch television (TV) at least once a week, less than once a week, or not at all? A binary composite variable for mass media exposure was then created by categorizing those who reported accessing all three media at least once a week, as exposed to mass media, while those who responded less than once a week or not at all were considered not exposed to mass media [39].

#### Covariates

The selection of covariates for this study was based on Andersen’s Behavioural Model of Health Services Use [40] and a review of the relevant literature [41–47]. The included variables were categorised as predisposing, enabling, and need factors (see Fig. 2).

Predisposing factors included age (30–39, 40–49 years), marital status (never, currently, and formerly married), education level (none, primary school, and secondary school or above), parity (nullipara, uniparous, multiparous), modern contraceptive use (yes, no), and history of HIV testing (yes or no).

Enabling factors included distance to health facility (big problem, not a big a problem), employment status (unemployed, employed), place of residence (rural, urban), and wealth index. The wealth index is computed using the principal component analysis (PCA), which assigns weights to various asset and dwelling characteristics and standardizes the resulting scores to a standard normal distribution. Further details are available elsewhere [48]. In the DHS, the index was originally categorized into five groups: poorest, poor, medium, rich, and richest. For this study, it was reclassified into three categories: poor (combining poorest and poor), medium, and rich (combining rich and richest) to simplify ordinal interpretation.

Need factors included self-rated health status (bad, moderate, good) and visit to health facility in the past 12 months (yes, no).

#### Data management and analysis

We extracted, cleaned, and analysed data from the DHS using Stata version 16 (Stata Corporation, College Station, TX, USA). To account for the complex survey design, we incorporated strata, cluster, and weighting variables in our analysis. The data were weighted to reflect the survey design and adjusted for the probability of selection, non-response, and post-stratification adjustments.

A series of descriptive analyses were conducted for each country. Subsequently, the datasets were appended to describe the pooled characteristics of all the study participants. The results were presented as weighted frequencies (n) and percentages (%). Crosstabulation using Pearson’s Chi-squared test of independence was used to assess the association between categorical variables. We then conducted univariate logistic regression to assess the relationship between mass media exposure and cervical cancer screening (Model 1). This was followed by a multivariable logistic regression (Model 2), which examined the association between mass media exposure and cervical cancer screening while controlling for the effects of covariates. To ensure the validity of the regression model, multicollinearity was assessed using variance inflation factors (VIFs). All covariates had VIF values below 10, indicating the absence of multicollinearity. A stratified analysis was also performed to assess the relationship between mass media exposure and cervical cancer screening by area of residence (rural vs. urban). Furthermore, we examined the association between exposure to each type of mass media (i.e., newspaper, radio, and TV) and cervical cancer screening while adjusting for covariates. Crude and adjusted odds ratios along with their respective 95% confidence intervals were estimated. Statistical significance was evaluated using a two-tailed alpha level set at 5%.

#### Ethics statement

We used publicly available datasets from the DHS, and no further ethical approval was required. Permission to use the data was granted by the DHS program team.

## Results

### Background characteristics of participants

Table 1 presents the characteristics of the participants included in the analysis. Over half of the participants (59.1%) were aged between 30 and 39 years. The majority were currently married (75.1%), multiparous (88.5%), and had been tested for HIV (84.9%). Most participants were employed (71.1%) and reported that the distance to health facilities was not a major issue (71.1%). Regarding self-rated health, 71.0% considered their health good. In terms of educational attainment, 41.4% had primary education. Approximately 57.2% resided in rural areas, 47.0% were classified as rich, and 33.7% were considered poor. Additionally, 56.9% had visited a health facility in the past 12 months, 34.4% used modern contraceptives, and only 3.3% were exposed to all three forms of mass media at least once a week.

**Table 1:** Characteristics of study participants by country and overall pooled data

Variable name	Ghana (*N* = 7106)	Kenya (*N* = 7635)	Mozambique (*N* = 5320)	Tanzania (*N* = 6875)	All countries (*N* = 26936)
	*n* (%)	*n* (%)	*n* (%)	*n* (%)	*n* (%)
Age					
30–39	4238 (59.1)	4668 (61.1)	3084 (58.4)	3909 (57.3)	15899 (59.1)
40–49	2868 (40.9)	2967 (38.9)	2236 (41.6)	2966 (42.7)	11037 (40.9)
Educational level					
No education	2471 (25.4)	1273 (7.9)	1768 (38.5)	1483 (21.7)	6995 (22.1)
Primary	1130 (15.8)	3492 (48.2)	2276 (40.3)	392 (62.1)	10,819 (41.4)
Secondary and higher	3505 (58.9)	2870 (43.9)	1276 (21.3)	1471 (16.1)	9122 (36.5)
Marital status					
Never married	407 (7.1)	427 (6.2)	205 (3.3)	355 (5.2)	1394 (5.6)
Currently married	5698 (75.9)	5753 (74.0)	3949 (75.7)	5246 (74.9)	20646 (75.1)
Formerly married	1001 (17.0)	1455 (19.8)	1166 (21.0)	1274 (19.9)	4896 (19.3)
Place of residence					
Urban	3472 (57.1)	2769 (38.8)	2263 (38.7)	2404 (35.0)	10908 (42.8)
Rural	3634 (42.9)	4866 (61.2)	3057 (61.3)	4471 (65.0)	16028 (57.2)
Household wealth status					
Poor	3273 (33.9)	3010 (32.6)	1440 (35.1)	2172 (33.6)	9895 (33.7)
Middle	1335 (19.3)	1543 (19.5)	1026 (18.7)	1432 (19.6)	5336 (19.3)
Rich	2498 (46.8)	3082 (47.9)	2854 (46.3)	3271 (46.8)	11705 (47.0)
Employment status					
Unemployed	820 (10.0)	2631 (29.4)	2738 (57.9)	1898 (26.4)	8087 (28.9)
Employed	6286 (90.0)	5004 (70.6)	2582 (42.1)	4977 (73.6)	18849 (71.1)
Tested for HIV					
No	2347 (27.5)	558 (4.7)	961 (23.2)	511 (7.4)	4377 (15.1)
Yes	4759 (72.5)	7077 (95.4)	4359 (76.8)	6364 (92.6)	22559 (84.9)
Self-rated health status					
Bad	358 (4.8)	280 (3.8)	143 (1.9)	97(1.4)	878 (3.1)
Moderate	1421 (20.9)	1827 (25.5)	1448 (28.9)	1988 (29.4)	6684 (25.9)
Good	5327 (74.3)	5528 (70.8)	3729 (69.2)	4790 (69.2)	19374 (71.0)
Distance to health facility					
Not a big problem	5227 (77.1)	5392 (74.0)	3365 (60.4)	4975 (69.7)	18959 (71.1)
Big problem	1879 (22.9)	2243 (26.0)	1955 (39.6)	1900 (30.3)	7977 (28.9)
Parity					
Nullipara	343 (6.0)	186 (2.6)	181 (3.4)	278 (3.6)	988 (3.9)
Uniparous	576 (9.2)	528 (8.2)	350 (6.3)	409 (6.2)	1863 (7.6)
Multiparous	6187 (84.8)	6921 (89.2)	4789 (90.3)	6188 (90.2)	24085 (88.5)
Modern contraceptive use					
No	5348 (75.0)	4096 (48.6)	3607 (72.4)	4884 (69.5)	17935 (65.6)
Yes	1758 (25.0)	3539 (51.4)	1713 (27.6)	1991 (30.5)	9001 (34.4)
Ever been screened for cervical cancer					
No	6652 (92.7)	5923 (73.0)	4426 (87.0)	6057 (87.7)	23058 (84.8)
Yes	454 (7.3)	1712 (27.0)	894 (13.0)	818 (12.3)	3878 (15.2)
Visit to health facility in the past 12 months					
No	3077 (44.0)	3510 (44.1)	1676 (39.5)	2944 (44.0)	11207 (43.1)
Yes	4029 (56.0)	4125 (55.9)	3644 (60.5)	3931 (56.0)	15729 (56.9)
Mass media exposure					
No	7001 (98.0)	7270 (94.4)	5195 (98.0)	6638 (96.9)	26104 (96.7)
Yes	105 (2.0)	365 (5.6)	125 (2.0)	237 (3.1)	832 (3.3)

### Prevalence of cervical cancer screening

**Fig. 3:**
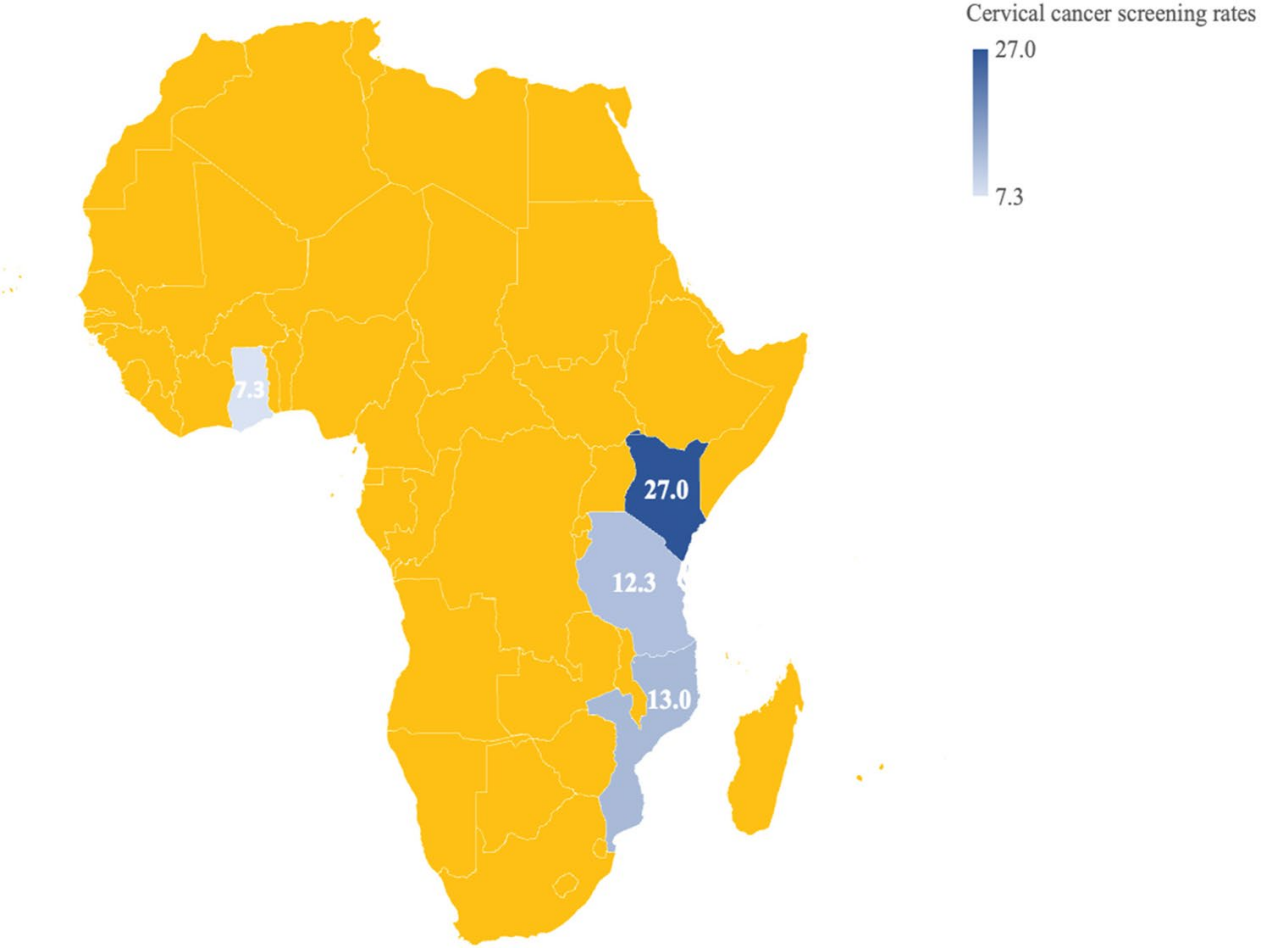
Prevalence of cervical cancer screening among women aged 30–49 years in four SSA countries. Countries shown in yellow were not included in the analysis

The pooled prevalence of cervical cancer screening among the study participants was 15.2% (Table 1). Kenya had the highest proportion of women screened (27.0%), followed by Mozambique (13.0%), Tanzania (12.3%) and Ghana (7.3%), as shown in Fig. 3.

### Association between mass media exposure and cervical cancer screening

As displayed in Table 2, the unadjusted analysis (Model 1) showed a significant positive association between mass media exposure and cervical cancer screening. Specifically, women exposed to mass media had more than three times the odds of being screened for cervical cancer (COR: 3.16, 95% CI: 2.57–3.89) compared to those not exposed. This association persisted even after adjusting for covariates (Model 2), with women exposed to mass media being 74% more likely to be screened for cervical cancer (AOR: 1.74, 95% CI: 1.40–2.16).

**Table 2:** Association between mass media exposure and cervical cancer screening among women aged 30–49 years in four SSA countries

Variable	Cervical cancer screening status		Mode1 COR (95% CI)	Model 2 ^a^AOR (95% CI)	*P*-value
	Not screened (*n* = 23058)	Screened (*n* = 3878)			
	*n* (%)	*n* (%)			
Media exposure					
No (Reference)	6561 (94.5)	403 (5.5)	1.00	1.00	
Yes	16497 (81.7)	3475 (18.3)	**3.16 (2.57–3.89)**	**1.74 (1.40–2.16)**	**< 0.001**

Significant values are in bold font

*COR* Crude odds ratio, *AOR* Adjusted odds ratio, *CI* confidence interval

^a^Adjusted for age, educational level, marital status, place of residence, wealth index status employment status, HIV testing, self-rated heath status, distance to health facility, parity, modern contraceptive use, and visited health facility in the past 12 months

### Association between mass media exposure and cervical cancer screening, stratified by area of residence

Table 3 shows the association between mass media exposure and cervical cancer screening among women aged 30–49 years, stratified by area of residence (rural vs. urban). Women exposed to mass media were more likely to undergo cervical cancer screening in urban areas (AOR: 2.02, 95% CI: 1.55–2.65) compared to those with no media exposure. However, in rural areas, mass media exposure was not significantly associated with screening uptake (AOR: 1.19, 95% CI: 0.84–1.68).

**Table 3:** Association between mass media exposure and cervical cancer screening among women aged 30–49 years in four SSA countries, stratified by area of residence

Variable	Residence	
	Rural	Urban
	^a^AOR (95% CI)	^a^AOR (95% CI)
Mass media exposure		
No (Reference)	1.00	1.00
Yes	1.19 (0.84–1.68)	**2.02 (1.55–2.65)**

Significant values are in bold font

*AOR* Adjusted odds ratio, *CI* confidence interval

^a^Adjusted for age, educational level, marital status, wealth index status employment status, HIV testing, self-rated heath status, distance to health facility, parity, modern contraceptive use, and visited health facility in the past 12 months

### Association between specific mass media exposure and cervical cancer screening

Table 4 shows that exposure to newspapers (AOR: 1.60, 95% CI: 1.34–1.90), radio (AOR: 1.27, 95% CI: 1.14–1.40), and TV (AOR: 1.16, 95% CI: 1.04–1.30) were all associated with higher cervical cancer screening rates compared to women who were not exposed to these media.

**Table 4:** Association between exposure to specific mass media and cervical cancer screening among women aged 30–49 years in four SSA countries

Mass media type		
	COR (95% CI)	^a^AOR (95% CI)
Exposure to newspaper		
No (Reference)	**1.00**	**1.00**
Yes	**2.92 (2.47–3.45)**	**1.60 (1.34–1.90)**
Exposure to radio		
No (Reference)	**1.00**	**1.00**
Yes	**1.90 (1.72–2.11)**	**1.27 (1.14–1.40)**
Exposure to TV		
No (Reference)	**1.00**	**1.00**
Yes	**2.46 (2.22–2.73)**	**1.16 (1.04–1.30)**

Significant values are in bold font.

*COR *Crude odds ratio,* AOR* Adjusted odds ratio, *CI* Confidence interval.

^a^Exposures to each type of media (TV, newspaper, and radio) were included simultaneously in the model to examine their independent associations with screening while adjusting for age, educational level, marital status, place of residence, wealth index, employment status, HIV testing, self-rated heath status, distance to health facility, parity, modern contraceptive use, visit to health facility in the past 12 months

## Discussion

We examined the association between mass media exposure and cervical cancer screening among women aged 30–49 years using DHS data from four SSA countries. After controlling for covariates, our analysis shows that women exposed to mass media had higher odds of undergoing screening than those not exposed, suggesting a positive association between mass media exposure and cervical cancer screening uptake.

Our finding aligns with previous studies that have reported a positive relationship between mass media exposure and health seeking behaviours, including cancer screening [49–51]. For instance, a study conducted in Australia found that exposure to a media campaign increased the number of women screened for cervical cancer by 27% [52]. Similarly, previous studies in Kenya reported that women exposed to mass media were more likely to screen for cervical cancer [34, 35]. Mass media play multiple roles by informing, educating, and entertaining people, which collectively contribute to promoting healthy behaviours [53]. In particular, media can shape public perceptions, increase knowledge about health issues, and inspire action, regarding preventive health measures like screening for cancer. Moreover, media can simplify complex ideas, reduce fear, and motivate women to seek preventive care by providing accurate and accessible information. Thus, mass media serve as a powerful tool in public health interventions due to their ability to reach diverse populations and deliver critical health messages.

Our results further indicate that mass media exposure was significantly associated with cervical cancer screening among women in urban areas but not in rural areas. Women in urban areas who were exposed to mass media were more likely to undergo screening than those without media exposure. However, this association was not observed in rural areas. These findings are in line with previous studies highlighting the influence of urban residence on screening uptake [54, 55]. The positive association between mass media exposure and screening uptake observed in urban areas may be partly attributed to better access to diverse media sources, higher literacy levels, and improved healthcare infrastructure, which may facilitate greater screening uptake. In contrast, rural areas often have limited access to media platforms due to socioeconomic barriers, infrastructural constraints, and lower literacy levels, which may reduce the reach and impact of health-related information. Our findings highlight the need for strategies to expand media reach in rural areas and complement mass media campaigns with community-based approaches to improve cervical cancer screening uptake.

We also observed that exposure to each type of mass media, including radio, TV, and newspapers, was independently associated with a higher likelihood of cervical cancer screening. This is consistent with the findings of a study in Côte d’Ivoire, which reported that women exposed to radio and newspapers had higher odds of screening for cervical cancer compared to those not exposed [36]. Similarly, a study conducted in five SSA countries found a positive association between these media sources and screening uptake [15], suggesting that these media platforms serve as key sources of health information, raising awareness about the importance of screening and influencing health-seeking behaviours. Radio is traditionally known as the primary source of information, especially in African countries [56]. It is widely accessible, even in rural areas, and often broadcasts health promotion messages that encourage preventive healthcare practices. Television provides visual content that can enhance understanding and motivation to seek screening services, while newspapers, though less useful to populations with low literacy levels, provide detailed health messages that may promote awareness and support informed decision-making. Additionally, women who frequently engage with these media platforms may have higher health literacy, making them more receptive to cervical cancer prevention messages.

### Implications for public health policy

These findings have important public health implications. The positive association observed between mass media exposure and cervical cancer screening suggests that improving access to media channels may support screening uptake in SSA. Since exposure to radio, television, and newspapers was associated with higher screening uptake, investing in these platforms could strengthen health communication, raise awareness about cervical cancer, promote preventive health behaviours, and ultimately increase screening rates, leading to earlier detection and improved health outcomes for women in SSA.

However, differences in the relationship between mass media exposure and screening uptake across urban and rural populations suggest the need for targeted strategies to ensure equitable access to health information. Expanding media outreach in rural areas through community radio programs, mobile health initiatives, and culturally tailored messaging could help close screening gaps. Additionally, integrating media campaigns with community-based interventions, such as healthcare worker outreach and peer education programs, may improve their effectiveness, particularly among populations with low literacy. Public health authorities should also collaborate with media organizations to develop accurate, engaging, and contextually relevant contents that address misconceptions and promote cervical cancer prevention in SSA.

### Strengths and limitations

The major strength of this study is the use of a large sample size pooled from DHS data across four SSA countries, which increased the statistical power of our analysis and allows the findings to be generalized to these four African countries. Additionally, the data were collected during similar periods, which enhances the validity of our findings. However, this study has some limitations. First, the cross-sectional design limits our ability to infer causality between media exposure and cervical cancer screening. Second, the variables were self-reported, making them prone to recall bias. Third, while mass media are widely used in many SSA countries to disseminate health messages, including those related to cancer prevention, the DHS does not include specific questions about exposure to cervical cancer-related content. As such, our measure of mass media exposure reflects general media consumption rather than targeted health communication on cervical cancer screening. Fourth, although we adjusted for several socioeconomic characteristics, these variables may not fully capture all the social or structural conditions that influence both media exposure and screening. Therefore, the observed association may partly reflect the effect of unmeasured or imperfectly measured factors. Lastly, the study did not include certain covariates, such as health insurance coverage, because they were not collected in some of the countries.

## Conclusion

This study shows a positive association between mass media exposure and cervical cancer screening among women aged 30–49 years in SSA, suggesting that increasing media access could improve screening rates and ultimately reduce cervical cancer morbidity and mortality in the region. Future research should explore causal relationships using longitudinal designs and investigate how cultural factors influence media effectiveness in promoting health-seeking behaviours.

## Supplementary Information

Supplementary material 1

## Data Availability

The datasets that support the findings of this study are publicly available from the Demographic and Health Surveys (DHS) Program. These datasets can be accessed upon reasonable request at https://dhsprogram.com/data/.

## Abbreviations

DHS: Demographic and Health Survey
HIV: Human Immunodeficiency Virus
HPV: Human Papillomavirus
LMICs: Low–and Middle–Income Countries
SSA: Sub-Saharan Africa
TV: Television
UNAIDS: United Nations programme on HIV/AIDS
UNFPA: United Nations Population Fund
UNICEF: United Nations Children’s Fund
USAID: United States Agency for International Development
VIA: Visual Inspection with Acetic Acid
WHO: World Health Organisation
