# Supplementary material for: Association between mass media exposure and cervical cancer screening uptake among women aged 30–49 years in four sub-Saharan African countries: a pooled analysis of demographic and health surveys
Source: BMC Womens Health. 2025 Dec 22;25:603. doi: 10.1186/s12905-025-04107-1 (PMC12723873; doi:10.1186/s12905-025-04107-1)
Supplement: Supplementary file 1 — Supplementary material 1 [file 12905_2025_4107_MOESM1_ESM.pdf]

STROBE Statement—Checklist of items that should be included in reports of ***cross-sectional studies***

	Item No	Recommendation
**Title and abstract**	1	(*a*) Indicate the study's design with a commonly used term in the title or the abstract
		(*b*) Provide in the abstract an informative and balanced summary of what was done and what was found This has been done
**Introduction**		
Background/rationale	2	Explain the scientific background and rationale for the investigation being reported Included
Objectives	3	State specific objectives, including any prespecified hypotheses Included
**Methods**		
Study design	4	Present key elements of study design early in the paper Included
Setting	5	Describe the setting, locations, and relevant dates, including periods of recruitment, exposure, follow-up, and data collection Included
Participants	6	(*a*) Give the eligibility criteria, and the sources and methods of selection of participants Included
Variables	7	Clearly define all outcomes, exposures, predictors, potential confounders, and effect modifiers. Give diagnostic criteria, if applicable Included
Data sources/ measurement	8*	For each variable of interest, give sources of data and details of methods of assessment (measurement). Describe comparability of assessment methods if there is more than one group Done
Bias	9	Describe any efforts to address potential sources of bias Done
Study size	10	Explain how the study size was arrived at Included
Quantitative variables	11	Explain how quantitative variables were handled in the analyses. If applicable, describe which groupings were chosen and why Included
Statistical methods	12	(*a*) Describe all statistical methods, including those used to control for confounding Done
		(*b*) Describe any methods used to examine subgroups and interactions Done
		(*c*) Explain how missing data were addressed Done
		(*d*) If applicable, describe analytical methods taking account of sampling strategy Included
		(*e*) Describe any sensitivity analyses Not Applicable
**Results**		
Participants	13*	(a) Report numbers of individuals at each stage of study—eg numbers potentially eligible, examined for eligibility, confirmed eligible, included in the study, completing follow-up, and analysed Done
		(b) Give reasons for non-participation at each stage Done
		(c) Consider use of a flow diagram
Descriptive data	14*	(a) Give characteristics of study participants (eg demographic, clinical, social) and information on exposures and potential confounders Included
		(b) Indicate number of participants with missing data for each variable of interest
Outcome data	15*	Report numbers of outcome events or summary measures Done
Main results	16	(*a*) Give unadjusted estimates and, if applicable, confounder-adjusted estimates and their precision (eg, 95% confidence interval). Make clear which confounders were adjusted for and why they were included Done
		(*b*) Report category boundaries when continuous variables were categorized N/A
		(*c*) If relevant, consider translating estimates of relative risk into absolute risk for a meaningful time period Done
Other analyses	17	Report other analyses done—eg analyses of subgroups and interactions, and sensitivity analyses Done

**Discussion**			
Key results	18	Summarise key results with reference to study objectives	Included
Limitations	19	Discuss limitations of the study, taking into account sources of potential bias or imprecision. Discuss both direction and magnitude of any potential bias	Done
Interpretation	20	Give a cautious overall interpretation of results considering objectives, limitations, multiplicity of analyses, results from similar studies, and other relevant evidence	Done
Generalisability	21	Discuss the generalisability (external validity) of the study results	Done
**Other information**			
Funding	22	Give the source of funding and the role of the funders for the present study and, if applicable, for the original study on which the present article is based	Included

*Give information separately for exposed and unexposed groups.

Note: An Explanation and Elaboration article discusses each checklist item and gives methodological background and published examples of transparent reporting. The STROBE checklist is best used in conjunction with this article (freely available on the Web sites of PLoS Medicine at http://www.plosmedicine.org/, Annals of Internal Medicine at http://www.annals.org/, and Epidemiology at http://www.epidem.com/). Information on the STROBE Initiative is available at www.strobe-statement.org.
